# *In vivo* evidence that RBM5 is a tumour suppressor in the lung

**DOI:** 10.1038/s41598-017-15874-9

**Published:** 2017-11-24

**Authors:** Duangporn Jamsai, D. Neil Watkins, Anne E. O’Connor, D. Jo Merriner, Selen Gursoy, Anthony D. Bird, Beena Kumar, Alistair Miller, Timothy J. Cole, Brendan J. Jenkins, Moira K. O’Bryan

**Affiliations:** 1 The School of Biological Sciences, Monash University, 25 Rainforest Walk, Clayton, Victoria, 3800 Australia; 2 The Development and Stem Cells Program of Monash Biomedicine Discovery Institute, 19 Innovation Walk, Clayton, Victoria, 3800 Australia; 3 Cancer Developmental Biology Group, The Garvan Institute of Medical Research, 384 Victoria St, Darlinghurst, Sydney, NSW 2010 Australia; 4 The Hudson Institute of Medical Research, Clayton, Victoria 3168 Australia; 5 Department of Anatomical Pathology, Monash Medical Centre, Monash Health, 246 Clayton Rd, Clayton, Victoria 3168 Australia; 6 General and Respiratory Medicine, Monash Medical Centre, Monash Health, 246 Clayton Rd, Clayton, Victoria 3168 Australia; 7 The Department of Biochemistry and Molecular Biology, Monash University, 19 Innovation Walk, Clayton, Victoria 3800 Australia; 8 Centre for Innate Immunity and Infectious Diseases, Hudson Institute of Medical Research, Monash University, 27-31 Wright St, Clayton, Victoria 3168 Australia

## Abstract

Cigarette smoking is undoubtedly a risk factor for lung cancer. Moreover, smokers with genetic mutations on chromosome 3p21.3, a region frequently deleted in cancer and notably in lung cancer, have a dramatically higher risk of aggressive lung cancer. The RNA binding motif 5 (RBM5) is one of the component genes in the 3p21.3 tumour suppressor region. Studies using human cancer specimens and cell lines suggest a role for RBM5 as a tumour suppressor. Here we demonstrate, for the first time, an *in vivo* role for RBM5 as a tumour suppressor in the mouse lung. We generated *Rbm5* loss-of-function mice and exposed them to a tobacco carcinogen NNK. Upon exposure to NNK, *Rbm5* loss-of-function mice developed lung cancer at similar rates to wild type mice. As tumourigenesis progressed, however, reduced *Rbm5* expression lead to significantly more aggressive lung cancer i.e. increased adenocarcinoma nodule numbers and tumour size. Our data provide *in vivo* evidence that reduced RBM5 function, as occurs in a large number of patients, coupled with exposure to tobacco carcinogens is a risk factor for an aggressive lung cancer phenotype. These data suggest that RBM5 loss-of-function likely underpins at least part of the pro-tumourigenic consequences of 3p21.3 deletion in humans.

## Introduction

Late stage detection makes lung cancer one of the most fatal forms of cancer, with a five-year survival rate of 17% overall, or below 2% for those with stage IV disease at diagnosis^1^. A 2012 World Health Organization report estimated that of the 1.8 million people diagnosed with lung cancer worldwide, 1.6 million would die from the disease^2^. However, if diagnosed in its earliest stages, surgery, chemotherapy and radiation therapy present a likely cure.

RNA binding motif 5 (*RBM5*) is one of the genes located within the tumour suppressor region 3p21.3; a region containing 19 genes that is frequently deleted in lung cancer and other types of carcinomas^3^. Moreover, the 3p21.3 deletion is detected in pre-neoplastic lesions in smokers^4^, indicating that it is an early change in the multistep pathogenesis of lung cancer. Despite this, a role for 3p21.3 deletions, and its component genes, in *in vivo* tumour development is still a matter of conjecture.

Human lung cancers can be divided into two main histopathological subtypes: non-small-cell lung cancer (NSCLC) and small-cell lung cancer (SCLC). NSCLC accounts for approximately 85% of lung cancers and can be divided into adenocarcinoma and squamous cell carcinoma (SCC). About 40% of human lung cancers are adenocarcinomas. Decreased *RBM5* expression, at the mRNA and protein levels, have been reported in primary NSCLC specimens compared to normal adjacent tissues^5^. Further, RBM5 is one of nine genes down-regulated in metastases of primary tumours^6^. RBM5 is also included in the 17 common gene signatures associated with metastasis identified in multiple solid tumour types. Solid tumours carrying this gene expression signature have higher rates of metastasis and poor clinical outcomes^6^. Decreased *RBM5* expression in primary lung tumours has been shown to correlate with lymph node metastasis^7^. In addition, RBM5 has been implicated in breast cancer development^8,9^, vestibular schwannomas^10^ and renal carcinomas^11^. Collectively, these data suggest that reduced *RBM5* expression is associated with increased cancer risk and that RBM5 is a tumour suppressor.

In support of this hypothesis, *in vitro* over-expression of RBM5 was shown to inhibit the growth of human lung cancer cell lines by increasing apoptosis and inducing cell cycle arrest in G1^12^. The inhibition of cell growth was associated with decreased cyclin A and phosphorylated retinoblastoma (RB) and an increase in the expression of the proapototic protein Bax^12^. RBM5 has been shown to suppress anchorage-dependent and anchorage-independent growth in A9 mouse fibrosarcoma cells and to inhibit their tumour forming activity in nude mice^5^.

RBM5 is an RNA binding protein that has previously been shown to regulate the splicing of apoptosis-related pre-mRNAs, including Caspase 2^13^, *FAS* receptor and *c-FLIP* ^14^, B-lymphocyte cytidine deaminase enzyme *AID* (activation-induced cytidine deaminase)^15^, Notch pathway regulator *Numb* in HeLa cells^16^ and numerous transcripts required for male germ cell development^17^. Its role as a splicing regulator is conserved in *Arabidopsis* ^18^.

Since the cloning of the *RBM5* gene^19^, a growing body of literature strongly suggests a role for RBM5 as a tumour suppressor^12–14,16^. However, the *in vivo* tumour suppression activity of RBM5 has not been tested. Within this study, we have generated *Rbm5* heterozygous knockout mice, i.e. analogous to the reduced expression seen in lung cancer patients, and have used them to demonstrate a role for RBM5 in *in vivo* tumour suppression function in the lung.

## Results and Discussion

**Figure 1:**
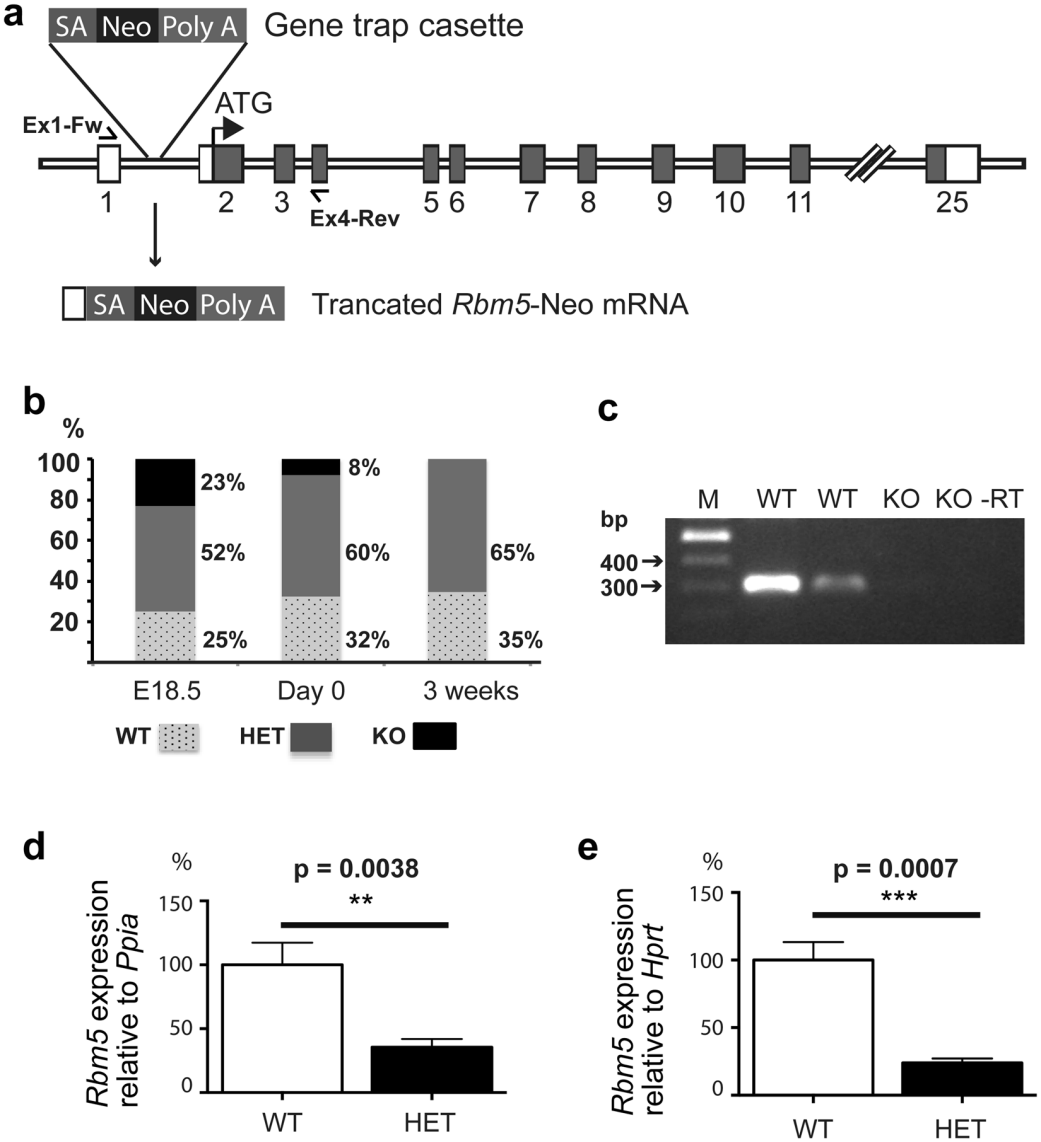
Generation and characterisation of the *Rbm5* gene trap mouse line. (**a**) The *Rbm5* gene trap mouse line: The U3neoSVFS gene-trapped cassette was inserted into intron 1 of the *Rbm5* gene (ENSMUSG00000032580). This produced a truncated *Rbm5* mRNA containing exon 1 (ENSMUSE00000371436), resulting in the production of truncated *Rbm5* mRNA and no protein production (null allele). SA: Splicing acceptor site; Neo: Neomycin resistance gene; Poly A: Polyadenylation signal. (**b**) Distribution of genotypes of progeny from heterozygous knockout breeding pairs at embryonic day 18.5 (E18.5), day of birth (Day 0) and at 3 weeks. WT: wild type; HET: heterozygous knockout; KO: homozygous knockout. (**c**) Verification of gene trapping efficient by RT-PCR on E18.5 lung using primers Ex1-Fw (5′-CTCCTGCTTTGTTCCCTCTG-3′) and Ex4-Rev (5′-CCATCTTCAGACCGGTCACT-3′). The WT allele expected PCR product is 298 bp and no products for the competed gene trap KO allele. -RT: negative control (no reverse transcriptase). (**d**,**e**) Quantitative PCR (qPCR) was performed to measure *Rbm5* mRNA expression levels in adult lung (D) and adult testis (E) samples. n = 3 per genotype, 8 weeks old. Data is expressed as mean ± SD. Statistical significance for all analyses was determined using a two-tailed student t-test.

To test the *in vivo* function of RBM5, we generated an *Rbm5* knockout mouse line using a gene trapped ES cell line. The gene-trapped cassette was inserted into intron 1 of the *Rbm5* gene. This produced a truncated *Rbm5* mRNA containing exon 1, resulting in no protein production (null allele) (Fig. 1a). Upon the establishment of a heterozygous knockout colony, the colony was backcrossed onto a C57BL6/J background for 10 generations. Mice heterozygous for the *Rbm5* knockout allele (referred to as HET, *Rbm5* ^+/−^) were viable, fertile and survived to adulthood with no detectable developmental defects. By contrast, and although a small number of homozygous *Rbm5* knockout mice (referred to as KO, *Rbm5* ^−/−^) were found, there was a pronounced deficit in *Rbm5* ^−/−^ pups on the day of birth i.e. 8%, compared to an expected frequency of 25%. There was an absence of *Rbm5* ^−/−^ mice at weaning age (0% at 3 weeks postnatal) (Fig. 1b). Indeed, virtually all *Rbm5* ^−/−^ pups were dead by 3 days post-natal. These data show that *Rbm5* is essential for either embryonic or early post-natal development. To define the time point when the embryo/foetus loss occurred, we collected pups from heterozygous time matings at E18.5. At this age, the ratio of each genotype was as expected according to the Mendelian rule (Fig. 1b). This finding indicates that *Rbm5* is absolutely required for the survival of newborn pups. The pathology underlying *Rbm5* ^−/−^ pup death is currently unknown.

In order to ascertain the degree of gene trapping efficiency, we collected lung tissues from wild type (WT, *Rbm5* ^+/+^) and *Rbm5* ^−/−^ foetuses at E18.5 and performed RT-PCR using primers flanking exons 1 and 4 (Fig. 1c). No *Rbm5* transcript was detected in the *Rbm5* ^−/−^ lungs compared to that of *Rbm5* ^+/+^ lungs. Furthermore, using quantitative PCR (qPCR) we showed that there was a significant reduction in the levels of *Rbm5* mRNA in the *Rbm5* ^+/−^ lung (64% reduction, Fig. 1d) and testis (76% reduction, Fig. 1e) collected from 8 week old mice. This data was mirrored using western blotting of adult lungs (Supplementary Fig. 1). This data indicate that the gene trap cassette was efficiently interrupting *Rbm5* gene expression as expected and that *Rbm5* ^+/−^ mice contained reduced *Rbm5* expression, analogous to the situation observed in many lung cancer patients.

**Figure 2:**
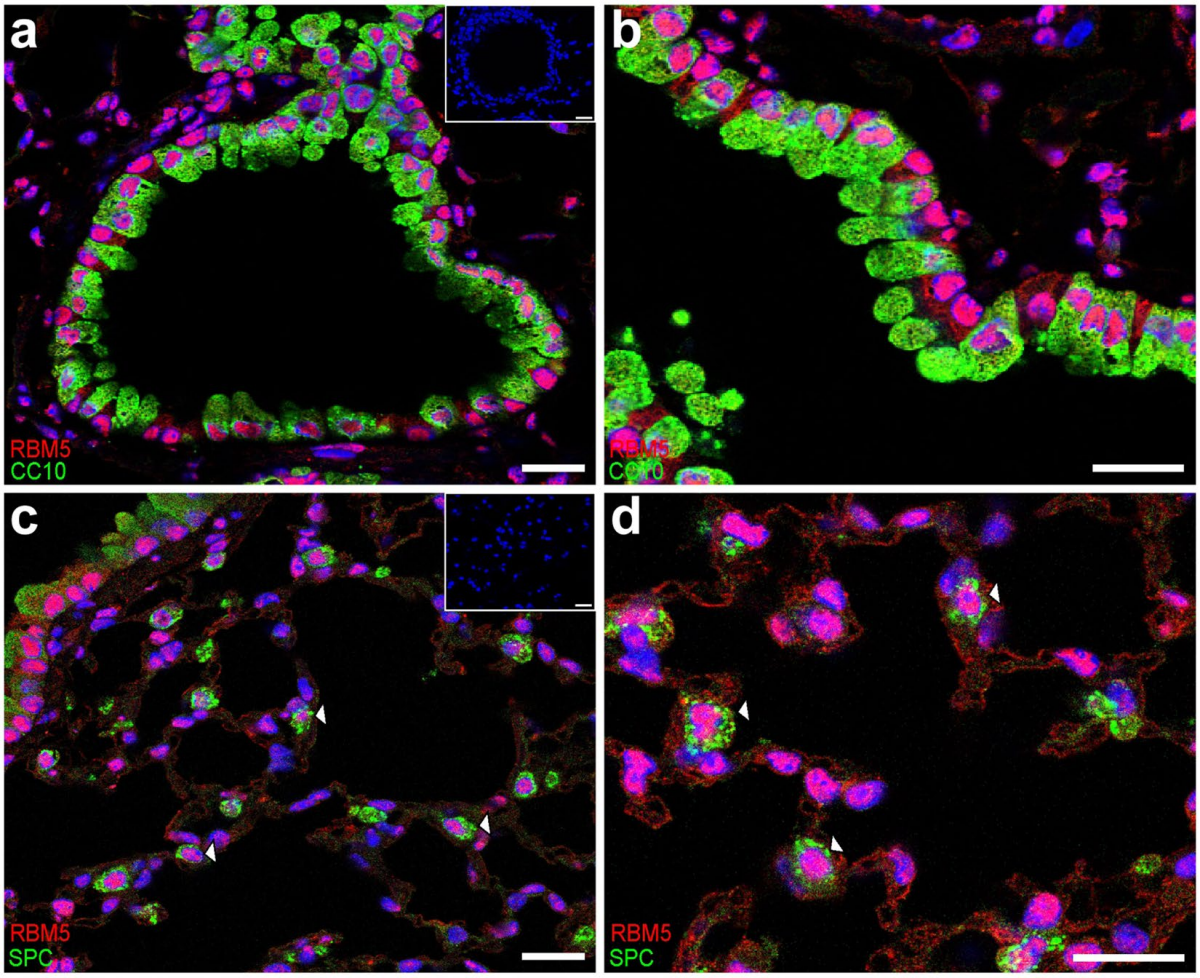
RBM5 localised to type II alveolar epithelial cells (AECs) and Clara cells. The localisation of RBM5 in the adult lung (8 weeks old) was determined by immunofluorescence using a RBM5 mouse monoclonal antibody as described previously^17^. (**a**,**b**) RBM5 localised to Clara cells as indicated by double staining for RBM5 (red) and CC10 (green). (**c**,**d** **)** RBM5 localised to type II alveolar epithelial cells (AECs) as indicated by double staining of RBM5 (red) and Pro-surfactant protein C (SPC, in green). Insets = negative controls (no primary antibody). Scare bars = 20 μm.

Based on mRNA expression pattern, *Rbm5* is a ubiquitously expressed gene with the highest expression level found in the testis^17^. Using immunofluorescence labelling, we showed that RBM5 protein was widely localized in the adult mouse lung. RBM5 was detected in almost all cells within the conducting airway epithelium, as shown by double-labelling with the secretory cell marker CC10 (also known as SCGB1A1) (Fig. 2a,b). RBM5 expression in CC10-positive and CC10-negative cells indicated localisation in both secretory and ciliated cells, respectively. In the distal lung, RBM5 expression was also detected in many cell lineages, including type II alveolar epithelial cells (AECs), as shown by double-labelling with the type-II AEC marker Pro-surfactant protein C (ProSPC) (Fig. 2c,d). A strong nuclear localisation was observed in both cell types. This result was consistent with previous studies demonstrating nuclear RBM5 localisation in male germ cells^17^ and HeLa cells^13^.

To date, the cellular origin of most types of lung cancers remained largely unknown. However, studies using a knock-in mouse model carrying a codon 12 *K-Ras* mutant gene indicate that type II AECs are the cells of origin of *K-Ras*-induced adenocarcinomas^20^. Given the localization of RBM5 to type II cells, and previous studies in cell lines and human tissue suggesting reduced RBM5 expression is associated with increased lung cancer risk^7,12^, we decided to test the susceptibility of reduced *Rbm5* expression (*Rbm5* ^+/−^) on lung cancer progression i.e. is ascertain if RBM5 is a tumour suppressor *in vivo*.

Lung cancer, like many other cancers, is thought to conform to the 2-hit model i.e. genetic and environmental hits. Cigarette smoking is the greatest risk factor for lung cancer^21^ and 4-(methylnitrosamino)-1-(3-pyridyl)-1-butanone (NNK) is the most potent carcinogen identified in cigarette smoke and has been widely used as accepted means of lung cancer induction^22^. As such, in this study, we utilized NNK to induce lung cancer in *Rbm5* heterozygous knockout (carrying one copy of the *Rbm5* gene) and wild type mice. The purpose of using NNK was to accelerate carcinogenesis into a time frame that would allow an in-depth analysis of lung cancer initiation and progression. Although there is a strong correlation between cigarette smoking and lung cancer, susceptibility to lung cancer among smokers is not uniform^23^. Smokers who carry particular genetic mutations have a dramatically higher risk of developing lung cancer^23^. Studies have shown that the *KRAS* activating mutation (G12V) is often associated with smoking-related NSCLC^24^. Similarly, different strains of mice have been shown to have differential susceptibility to lung cancer induction upon exposure to NNK^25^. The A/J mouse strain, that carries a naturally occurring *KRAS* G12V mutation, is one of the most susceptible strains for NNK-induced lung cancer. Importantly, RBM5 has been found to be down-regulated in rat embryonic fibroblast cells that have been constitutively over-expressed RAS G12V protein^26^. These studies suggest a correlation between RBM5 and KRAS activating mutation in the pathogenesis of lung cancer.

Given this association, to investigate the role of RBM5 in lung cancer, *Rbm5* ^+/−^ mice were backcrossed onto the A/J mouse strain for 10 generations. Six week-old *Rbm5* ^+/−^ (HET) and *Rbm5* ^+/+^ (WT) littermates were injected with NNK to induce lung cancer as previously described^27^. The use of *Rbm5* ^+/−^ mice (rather than *Rbm5* ^−/−^ mice) is reflective of the situation in patients wherein chromosome 3p21.3 loss occurs in heterozygosity. Lung tissues were analysis for the presence of tumours and the progression of tumour formation at 16, 20 and 48 weeks post-NNK injection.

At 16 and 20 weeks post-NNK injection, a small number of lung tumours were observed in both *Rbm5* ^+/+^ and *Rbm5* ^+/−^ mice, however no significant difference in tumour number or tumour area was observed (Supplementary Fig. 2a–d). The control group, which received saline injection, showed no sign of lung tumours (*n* = 10). This finding suggested that RBM5 dosage does not affect the initiation of NNK-induced lung cancer and is consistent with previously published data showing that NNK in isolation can induce lung cancer^28^. While not examined at molecular detail here, the histopathological progression of NNK-induced lung cancer progression was consistent with previous publications^28^.

**Figure 3:**
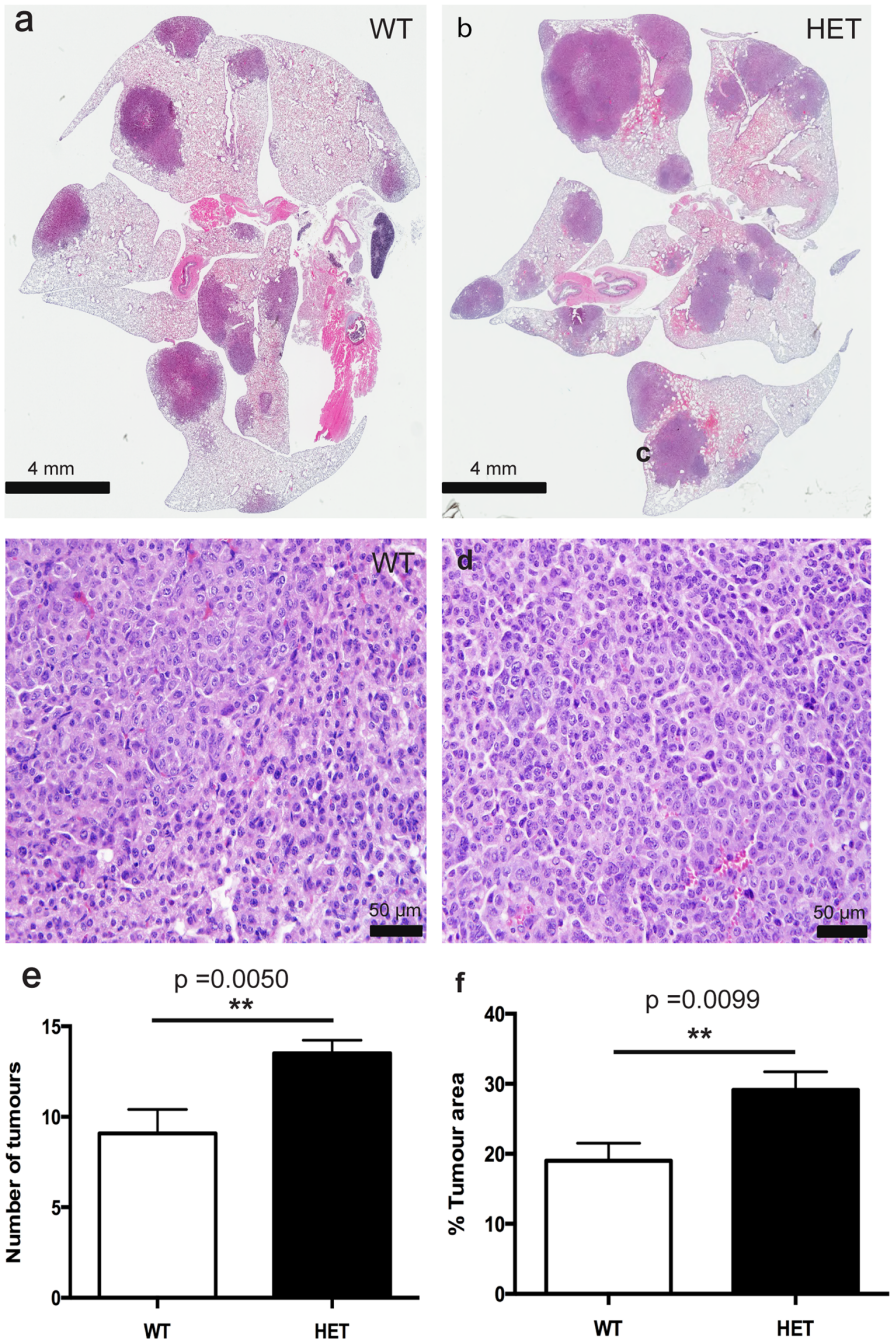
*Rbm5* haploinsufficiency leads to accelerated lung cancer progression. (**a**–**d**) H&E staining of lungs collected from mice 48 weeks post-NNK injection. Number of tumours (**e**) and tumour area (**f**) in mice 48 weeks post-NNK injection. Data are expressed as mean +/− S.E.M. (standard error of mean). *n* = 13 WT and *n* = 16 HET. p < 0.05 was considered statistically significance. Statistical significance for all analyses was determined using a two-tailed student t-test.

At 48 weeks post-NNK injection, however, *Rbm5* ^+/−^ mice displayed a significant increase in both the number of individual tumours, and tumour area, compared to *Rbm5* ^+/+^ mice (Fig. 3). An independent, and blinded, analysis by a pathologist confirmed that the *Rbm5* ^+/−^ lungs contained an increased numbers of adenocarcinoma nodules with increased tumour nodule sizes compared to that in *Rbm5* ^+/+^ lungs (148% and 153% increase on control wild type tissue respectively) (Fig. 3, Supplementary Table 1). These data indicate that reduced *Rbm5* expression leads to more aggressive progression of lung adenocarcinomas in the *Rbm5* ^+/−^ mice.

Consistent with previous publications suggesting at AEC cells are the origin of many lung cancers^20^, tumours in both *Rbm5* ^+/+^ and *Rbm5* ^+/−^ mice were Pro-SPC positive, and CC10 negative (Supplementary Fig. 3). Somewhat surprisingly, however, there was no significant difference in either the rates of apoptosis (TUNEL staining, data not shown) or Ki67 labelling with tumours between genotypes (Ki67: 186.5 +/− 59.92 in wild type versus 149.0 +/− 51.56 positive cells per mm^2^ in *Rbm5* ^+/−^). The rates of apoptosis per tumour area were too low to reliably quantitate in both genotypes. This data is perhaps, however, consistent with the long progression time required to see a difference in tumour mass between genotypes.

Lung cancer, like many other types of cancer, is believed to be initiated by over-activation of oncogenes and/or down-regulation of tumour suppressor genes. Although several studies using cell lines have provided evidence for RBM5 being a regulator of apoptosis and the cell cycle, direct *in vivo* evidence for its tumour suppressor activity remained elusive. Our data is the first to demonstrate the physiological role for RBM5 as a tumour suppressor in the lung. At 16–20 weeks post-exposure to NNK, we showed that reduced *Rbm5* expression had no effect of the initiation of lung adenocarcinomas. As tumourigenesis progressed, however, reduced *Rbm5* expression resulted in more aggressive tumour growth in *Rbm5* ^+/−^ lungs. This data is consistent with previous studies showing that decreased *RBM5* expression in human lung tumours is correlated with lymph node metastasis^7^, and ectopic over-expression of RBM5 inhibited tumour forming activity in nude mice^5^.

Metastatic lung cancer is a leading cause of mortality. Surgery, chemotherapy, and radiation therapy present a likely cure if the cancer is diagnosed in its earliest stages. The data presented herein suggests that a quantitative assessment of RBM5 expression in lung cancer biopsies, for example, may be a valuable prognostic marker for the prediction of high-risk cases for whom more aggressive post-surgical treatments may be warranted. Moreover, our data reveal that RBM5 is required for early post-natal survival and acts *in vivo* as a tumour suppressor that likely underpins at least part of the pro-tumourigenic outcomes resulting from 3p21.3 deletion in humans.

## Methods

### Mouse line production and genotyping

Animal procedures were conducted in accordance with the Australian National Health and Medical Research Council’s Guidelines on Ethics in Animal Experimentation and were approved by the Monash University Animal Experimentation Ethics Committee. The *Rbm5* gene trap mouse line was generated at the Australian Phenomics Network (APN) Monash University Node using standard methods^29^ and a gene-trapped ES cell (PST20293-NR, on a 129Sv x C57BL/6J background) obtained from the Toronto Centre for Phenogenomics Centre for Modelling Human Disease. The U3neoSVFS gene-trapped cassette was inserted into intron 1 of the *Rbm5* gene (ENSMUSG00000032580). This produced a truncated *Rbm5* mRNA containing exon 1 (ENSMUSE00000371436), resulting in the production of truncated *Rbm5* mRNA and no protein production (null allele).

Mouse genotypes were determined from tail biopsies using real time PCR with specific probes designed for each allele (Transnetyx, Cordova, TN). The *Rbm5* KO allele was detected using the Neomycin probe and primers set which included: Forward primer: GGGCGCCCGGTTCTT; Reporter: ACCTGTCCGGTGCCC; and Reverse primer: CCTCGTCCTGCAGTTCATTCA. The *Rbm5* WT allele was detected using the *Rbm5* WT probe set which included: Forward primer: CATTACACCCCAGTGATTTTGCA; reporter: TTGGTGCTGTCCCTTAAGTC; and Reverse primer: CCTCTGGCGGCTGACA.

Verification of gene trapping efficient was performed by RT-PCR on E18.5 lung using primers Ex1-Fw (5′-CTCCTGCTTTGTTCCCTCTG-3′) and Ex4-Rev (5′-CCATCTTCAGACCGGTCACT-3′). The WT allele expected PCR product is 298 bp and no products for the competed gene trap KO allele.

Quantitative PCR (qPCR) was performed to measure *Rbm5* mRNA expression levels in adult lung and adult testis samples (n = 3 per genotype, 8 weeks old) using TaqMan assays (*Rbm5* exons 4-5: Mm00455721, Thermo Scientifics). The primers used in the as The levels of *Rbm5* mRNA expression between *Rbm5* ^+/+^ and *Rbm5* ^+/−^ lung and testis samples were normalised against mRNA levels of *Ppia* (Mm02342429) and *Hprt* (Mm00446968), respectively.

At the time of writing this manuscript, data contained within Ensembl indicated that the mouse *Rbm5* gene (entry ENSMUSG00000032580) produced 24 transcripts, 7 of which are predicted to be protein coding and another 4 which are predicted to be subject to nonsense-mediated RNA decay. The remaining 13 splice variants contain retained introns and are of undefined significance. Primers against exons 4–5 will detect 5 of the 7 predicted protein coding isoforms and an additional 6 of the transcripts which are thought to undergo non-sense mediated decay.

### Western blotting

In order to assess the degree of protein reduction in *Rbm5* ^+/−^ mice, lung tissue was from wild type and *Rbm5* ^+/−^ adult mice then processed for western blotting as described previously^30^. Blots were probed using the RBM5 monoclonal antibody A9 described in^17^. The A9 antibody is predicted to be able to bind to all 7 of the protein coding isoforms, and would also bind to 3 of the 4 transcripts/proteins predicted to be subject to nonsense-mediated RNA decay if protein was to be produced.

### Immunochemical labelling of cells

The localisation of RBM5 in the adult lung (8 weeks old) was determined by immunofluorescence using an RBM5 mouse monoclonal antibody as described previously^17^. To distinguish different cell lineages in the lung, we co-labelled sections with antibodies against Pro-surfactant protein C (AB3786, Merck Millipore), as a marker for type II alveolar epithelial cells, and CC10 (CC10 (T-18), SC-9772, Santa Cruz Biotechnology), as a marker for secretory cells. Protein localisation was determined through confocal microscopy using an SP-8 microscope (Leica Microsystems).

### Immunochemical labelling of cells

In order to label cells undergoing apoptosis sections were staining using the TUNEL kit (ApopTag Peroxidase *In Situ* Apoptosis Detection Kit, Merck,S71000) as per the manufacturer’s instructions. In order to label proliferating cells, additional sections were labelled for Ki67 (NCL-Ki67p, Novacastra, at a dilution of 1 in 1000) using using immunohistochemistry. Bound antibody was detected using the Dako polymer, anti-rabbit, HRP kit as per the manufacturer’s instructions. The number of proliferating cells was subsequently quantitated from scanned slides using an Aperio ePathology scanner (Leica Biosystems) and Imagescope software. The number of apoptotic cells per tumour was too low to reliably quantitate. N = 6 samples per genotype were analysed. For both sets of labelling wild type tissue was used as a positive control.

### Lung cancer induction

NNK (Sapphire Biosciences, Cat. No. 000–01622) was used to induce lung tumours. Six-week old *Rbm5* ^+/−^ (HET) and *Rbm5* ^+/+^ (wild type, WT) mice were administered three i.p. injections over one week (Monday, Wednesday, Friday) at a dose of 50 mg/kg body weight. Mice were separated into two groups and were either injected with NNK (in saline, 0.1 ml volume) or vehicle (saline, 0.1 ml volume). Mice were humanely killed at 16, 20 and 48 weeks after the final NNK injection. Mice were anaesthetised, an incision was made in the trachea then lungs were perfusion fixed via a tracheal cannula with 4% formaldehyde at exactly 200 mm H_2_O pressure. Fixed tissues were paraffin embedded, sectioned and used for histological analysis (hematoxylin and eosin (H&E) staining). Total tumour area was measured using Imagescope software (Apeiro).

Data analysis was performed using GraphPad Prism 6 software and p < 0.05 was considered statistically significance. Histopathology was independently assessed in a blinded manner by a qualified pathologist (BK).

## Electronic supplementary material

Supplementary information

## References

[1] Ettinger DS (2015). Non-small cell lung cancer, version 6.2015. Journal of the National Comprehensive Cancer Network: JNCCN.

[2] Ferlay J (2015). Cancer incidence and mortality worldwide: sources, methods and major patterns in GLOBOCAN 2012. International journal of cancer. Journal international du cancer.

[3] Angeloni D (2007). Molecular analysis of deletions in human chromosome 3p21 and the role of resident cancer genes in disease. Briefings in functional genomics & proteomics.

[4] Kok K, Naylor SL, Buys CH (1997). Deletions of the short arm of chromosome 3 in solid tumors and the search for suppressor genes. Advances in cancer research.

[5] Oh JJ, West AR, Fishbein MC, Slamon DJ (2002). A candidate tumor suppressor gene, H37, from the human lung cancer tumor suppressor locus 3p21.3. Cancer research.

[6] Ramaswamy S, Ross KN, Lander ES, Golub TR (2003). A molecular signature of metastasis in primary solid tumors. Nature genetics.

[7] Oh JJ (2010). RBM5/H37 tumor suppressor, located at the lung cancer hot spot 3p21.3, alters expression of genes involved in metastasis. Lung cancer.

[8] Rintala-Maki ND (2007). Expression of RBM5-related factors in primary breast tissue. Journal of cellular biochemistry.

[9] Rintala-Maki ND, Abrasonis V, Burd M, Sutherland LC (2004). Genetic instability of RBM5/LUCA-15/H37 in MCF-7 breast carcinoma sublines may affect susceptibility to apoptosis. Cell biochemistry and function.

[10] Welling DB, Lasak JM, Akhmametyeva E, Ghaheri B, Chang LS (2002). cDNA microarray analysis of vestibular schwannomas. Otology & neurotology: official publication of the American Otological Society, American Neurotology Society [and] European Academy of Otology and Neurotology.

[11] Scanlan MJ (1999). Antigens recognized by autologous antibody in patients with renal-cell carcinoma. International journal of cancer. Journal international du cancer.

[12] Oh JJ (2006). 3p21.3 tumor suppressor gene H37/Luca15/RBM5 inhibits growth of human lung cancer cells through cell cycle arrest and apoptosis. Cancer research.

[13] Fushimi K (2008). Up-regulation of the proapoptotic caspase 2 splicing isoform by a candidate tumor suppressor, RBM5. Proceedings of the National Academy of Sciences of the United States of America.

[14] Bonnal S (2008). RBM5/Luca-15/H37 regulates Fas alternative splice site pairing after exon definition. Molecular cell.

[15] Jin W, Niu Z, Xu D, Li X (2012). RBM5 promotes exon 4 skipping of AID pre-mRNA by competing with the binding of U2AF65 to the polypyrimidine tract. FEBS letters.

[16] Bechara EG, Sebestyen E, Bernardis I, Eyras E, Valcarcel J (2013). RBM5, 6, and 10 differentially regulate NUMB alternative splicing to control cancer cell proliferation. Molecular cell.

[17] O’Bryan MK (2013). RBM5 is a male germ cell splicing factor and is required for spermatid differentiation and male fertility. PLoS genetics.

[18] Sugliani M, Brambilla V, Clerkx EJ, Koornneef M, Soppe WJ (2010). The conserved splicing factor SUA controls alternative splicing of the developmental regulator ABI3 in Arabidopsis. The Plant cell.

[19] Oh JJ, Grosshans DR, Wong SG, Slamon DJ (1999). Identification of differentially expressed genes associated with HER-2/neu overexpression in human breast cancer cells. Nucleic acids research.

[20] Xu X (2012). Evidence for type II cells as cells of origin of K-Ras-induced distal lung adenocarcinoma. Proceedings of the National Academy of Sciences of the United States of America.

[21] Walser T (2008). Smoking and lung cancer: the role of inflammation. Proceedings of the American Thoracic Society.

[22] Zheng HC, Takano Y (2011). NNK-Induced Lung Tumors: A Review of Animal Model. Journal of oncology.

[23] Hecht SS (1999). Tobacco smoke carcinogens and lung cancer. Journal of the National Cancer Institute.

[24] Pao W (2005). KRAS mutations and primary resistance of lung adenocarcinomas to gefitinib or erlotinib. PLoS medicine.

[25] Razani-Boroujerdi S, Sopori ML (2007). Early manifestations of NNK-induced lung cancer: role of lung immunity in tumor susceptibility. American journal of respiratory cell and molecular biology.

[26] Edamatsu H, Kaziro Y, Itoh H (2000). LUCA15, a putative tumour suppressor gene encoding an RNA-binding nuclear protein, is down-regulated in ras-transformed Rat-1 cells. Genes to cells: devoted to molecular & cellular mechanisms.

[27] Miller, A., Brooks, G. D., McLeod, L., Ruwanpura, S. & Jenkins, B. J. Differential involvement of gp130 signalling pathways in modulating tobacco carcinogen-induced lung tumourigenesis. *Oncogene* **0**, 10.1038/onc.2014.99 (2014). 10.1038/onc.2014.99 24727895

[28] Ge GZ, Xu TR, Chen C (2015). Tobacco carcinogen NNK-induced lung cancer animal models and associated carcinogenic mechanisms. Acta biochimica et biophysica Sinica.

[29] Cotton LM (2015). Utilising the resources of the International Knockout Mouse Consortium: the Australian experience. Mammalian genome: official journal of the International Mammalian Genome Society.

[30] Okuda H (2017). LRGUK1 is part of a multiprotein complex required for manchette function and male fertility. FASEB journal: official publication of the Federation of American Societies for Experimental Biology.

