# Supplementary material for: In vivo evidence that RBM5 is a tumour suppressor in the lung
Source: Sci Rep. 2017 Nov 24;7:16323. doi: 10.1038/s41598-017-15874-9 (PMC5701194; doi:10.1038/s41598-017-15874-9)
Supplement: Supplementary file 1 — Supplementary information [file 41598_2017_15874_MOESM1_ESM.pdf]

*In vivo* evidence that RBM5 is a tumour suppressor in the lung

Duangporn Jamsai[1], D. Neil Watkins[2], Anne E. O'Connor[1], D. Jo Merriner[1], Selen Gursoy[1], Anthony D. Bird[3], Beena Kumar[4], Alistair Miller[5], Timothy J. Cole[6], Brendan J. Jenkins[7], Moira K. O'Bryan[1,*]

**Supplementary Table 1: Pathology report of lung samples collected at 48 weeks post-NNK injection**

| Reference number | Genotype | Number of tumour nodules | Dimension of tumour nodules (mm) | Tumour type |
|---|---|---|---|---|
| 10.199 | WT | 1 | 1 | Adenocarcinoma |
| 10.205 | WT | 2 | 0.3-0.8 | Adenocarcinoma |
| 10.206 | WT | 4 | 0.5-1 | Adenocarcinoma |
| 10.200 | WT | 6 | 0.3-1 | Adenocarcinoma |
| 10.210 | WT | 5 | 1.0-4 | Adenocarcinoma |
| 10.208 | HET | 7 | 1-5 | Adenocarcinoma |
| 10.198 | HET | 9 | 2-6 | Adenocarcinoma |
| 10.203 | HET | 9 | 0.5-5 | Adenocarcinoma |
| 10.201 | HET | 10 | 0.5-3 | Adenocarcinoma |
| 10.220 | HET | 14 | 0.2-3 | Adenocarcinoma |
| 10.221 | HET | 14 | 0.5-4 | Adenocarcinoma |

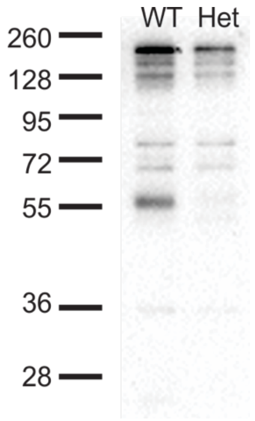


**Supplementary Figure 1:** A western blot illustrating the reduction in RBM5 protein in *Rbm5*$^{+/-}$ adult lung tissue compared to the corresponding *Rbm5*$^{+/+}$ tissue.

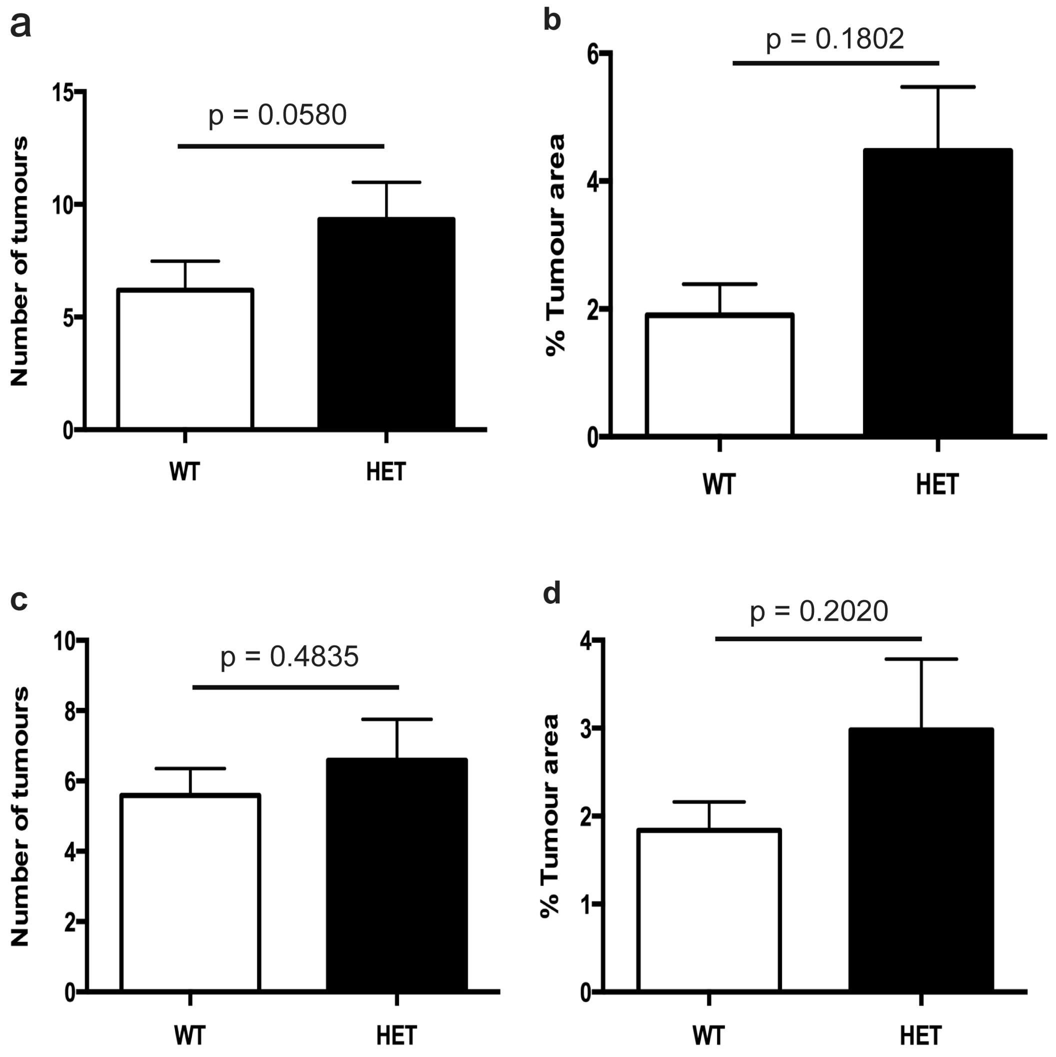


**Supplementary Figure 2: *Rbm5* is not critical for the initiation of NNK-induced lung cancer**

(A-B) Lung tumour analysis of *Rbm5*$^{+/-}$ (HET) and *Rbm5*$^{+/+}$ (WT) mice at 16 weeks post-NNK injection. n = 5 for WT and n = 6 for HET. (C-D) Lung tumour analysis of *Rbm5*$^{+/-}$ (HET) and *Rbm5*$^{+/+}$ (WT) mice at 20 weeks post-NNK injection. n = 12 for each genotype. No significant difference was observed at these two time points indicating that *Rbm5* is not critical for the formation of NNK-induced lung cancer.

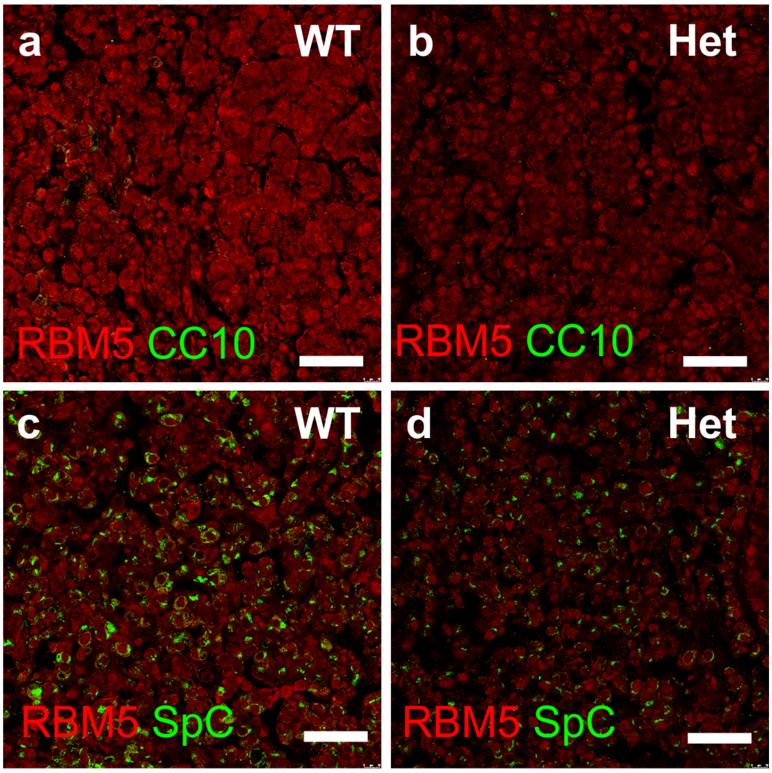


**Supplementary Figure 3:** Immunolabelling of tumour tissue within 48 weeks post-NNK injected wild type (WT) and *Rbm5*$^{+/-}$ (het) mice for RBM5 (red) and CC10 (green panels A-B) and Pro-SPC (Spc, green, panels C-D). Scale bar equals 50µm.
